# The silk of gorse spider mite *Tetranychus lintearius* represents a novel natural source of nanoparticles and biomaterials

**DOI:** 10.1038/s41598-020-74766-7

**Published:** 2020-10-28

**Authors:** Antonio Abel Lozano-Pérez, Ana Pagán, Vladimir Zhurov, Stephen D. Hudson, Jeffrey L. Hutter, Valerio Pruneri, Ignacio Pérez-Moreno, Vojislava Grbic’, José Luis Cenis, Miodrag Grbic’, Salvador Aznar-Cervantes

**Affiliations:** 1 Departmento de Biotecnología, Genómica y Mejora Vegetal, IMIDA, C/Mayor, s/n, 30150 La Alberca, Murcia, Spain; 2 Department of Biology, The University of Western Ontario, London, ON N6A 5B7 Canada; 3 Department of Physics and Astronomy, The University of Western Ontario, London, ON N6A 3K7 Canada; 4 ICFO—Institut de Ciències Fotòniques, The Barcelona Institute of Science and Technology, 08860 Castelldefels, Barcelona Spain; 5 Department of Agriculture and Food, University of La Rioja, C/Madre de Dios, 53, 26006 Logroño, La Rioja Spain; 6 Department of Biology, University of Belgrade, Belgrade, Serbia

**Keywords:** Biomedical materials, Biomaterials - proteins

## Abstract

Spider mites constitute an assemblage of well-known pests in agriculture, but are less known for their ability to spin silk of nanoscale diameters and high Young’s moduli. Here, we characterize silk of the gorse spider mite *Tetranychus lintearius*, which produces copious amounts of silk with nano-dimensions. We determined biophysical characteristics of the silk fibres and manufactured nanoparticles and biofilm derived from native silk. We determined silk structure using attenuated total reflectance Fourier transform infrared spectroscopy, and characterized silk nanoparticles using field emission scanning electron microscopy. Comparative studies using *T. lintearius* and silkworm silk nanoparticles and biofilm demonstrated that spider mite silk supports mammalian cell growth in vitro and that fluorescently labelled nanoparticles can enter cell cytoplasm. The potential for cytocompatibility demonstrated by this study, together with the prospect of recombinant silk production, opens a new avenue for biomedical application of this little-known silk.

## Introduction

Development of modern technologies relies heavily on novel materials with applications in a wide range of fields, including medicine and pharmacology, food production, engineering, and catalysis. Specific characteristics of materials necessary for particular applications include properties such as mechanical strength, elasticity, biocompatibility, biodegradability, size, density, and a combination of biochemical and physical characteristics that are often found in biological materials. Materials such as spider and silkworm silk have served as a basis for development of specific materials used in pharmacology, diagnostics, and regenerative medicine^1–3^, but have also inspired the development of biomimetic synthetic materials^4–6^.

Nanomaterials are emerging as an important element for modern therapeutic treatments. For effective drug delivery, potential nano-carriers must be biocompatible, biodegradable, non-toxic, and non-immunogenic, and need to allow versatile conjugation of specific drug load and delivery in a particular cellular compartment^7–9^. Currently, there are numerous nanomaterials on the market with often confusing nomenclature, such as “natural” biomaterials that can be of organic origin (spider or silkworm silk) or inorganic origin (titanium dioxide, nano-silver and many others), synthetic biomimetic materials (polymers, plastic), and materials produced by recombinant-technology. However, application of nanomaterials in modern life has raised concerns about their environmental safety and potential for adverse effects on human health^10^. Indeed, it has been shown that many engineered nanomaterials cause undesired effects in living organisms. For example, TiO_2_ affects circadian rhythm, immune and inflammatory response and basal metabolism, SiO_2_ affects immune response and genes involved in inflammation processes, and polystyrene nanomaterials are associated with effects on apoptosis, inflammation and basal metabolism^11^. Thus, biomaterials of natural origin are believed to have superior characteristics relative to synthetic materials. They are often biocompatible and biodegradable, and, as proteinaceous molecules, can be easily functionalized for specific applications. Silks are a family of proteinaceous materials secreted by many arthropods for different biological functions. The most studied examples of such organic materials are spider silk and silkworm silk. However, recent studies have uncovered “silk-like” materials from other arthropods including numerous insect species^12^, mites^13^ and even some marine species such as mussels^14^ and barnacles^15^. All these silk proteins, encoded by fibroin genes, contain common structural motifs, such as the β-pleated sheet, generating unique silk properties depending on slight differences in individual sequences. The majority of these naturally produced silk threads are in in the micron range of fibre thickness and are used for construction of cocoons, prey capturing, egg sac production, adherence to substrates or forming the pedicel of eggs^16^. Mechanical properties of these silk fibres can be expressed in terms their Young’s modulus, which characterizes the deformation due to applied stress, and ranges from 7 GPa in silkworm to 13.5 GPa in the spider *Nephila clavipes* to a high Young’s modulus of 28 GPa in the bugworm *Eumeta variegate*^17^.

**Figure 1:**
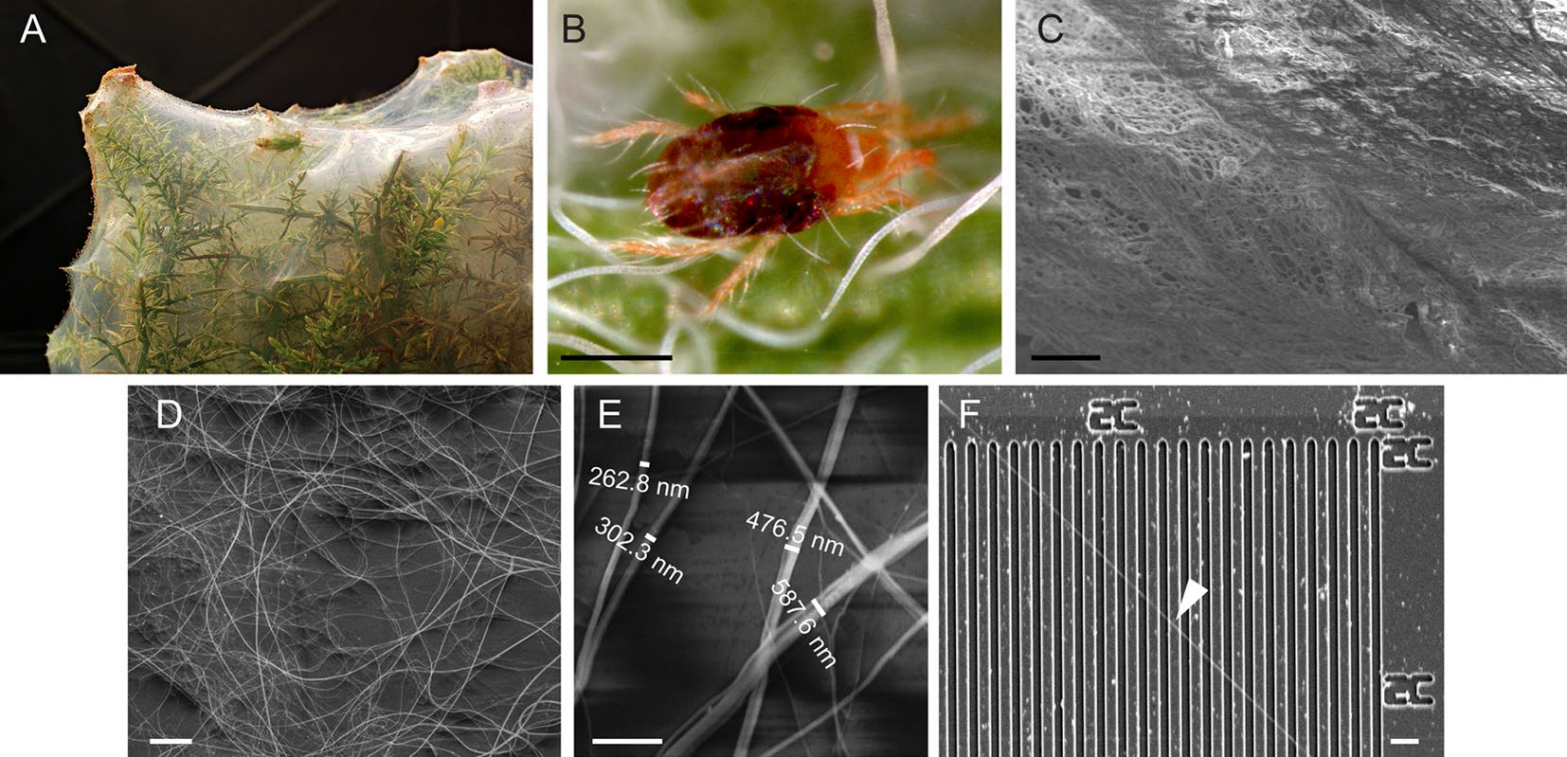
*T. lintearius* silk. (**A**) Gorse plant covered with thick “film” of *T. lintearius* silk; (**B**) adult *T. lintearius*; (**C**) native silk taken directly from the plant and visualised by SEM; (**D**) low density *T. lintearius* silk visualised by SEM; (**E**) measurements of individual silk fibers (SEM); (**F**) individual silk fiber (arrowhead) deposited on a silicon slide used for AFM measurements. Scale bar: b—100 µm, c, d and f—10 µm, e—2 µm.

Another little studied group that spins silk are phytophagous spider mites (*Tetranychide*). They belong to *acari*, in which the majority of species do not produce silk; however, spider mites from genus *Tetranychus* have evolved production of a versatile silk used for locomotion and dispersal from plant to plant^18–20^, protection from predators^18,20,21^, sheltering from climatic conditions^22^, as a surface for egg laying^23^, and for communication via pheromone and social-clue deposition^24,25^. Silk fibres from *Tetranychus urticae* have a striking characteristic: they have diameters on the nanometre scale, representing the thinnest natural silk fibre produced by silk spinning arthropods^13,26,27^. This nano-silk displays an extraordinarily high Young’s modulus that is almost double that of spider *N. clavipes* silk^13,26^ and is in the range of bagworm silk^17^, potentially representing a natural nanobiomaterial with valuable characteristics for technology and medicine. The *T. urticae* genome sequencing project allowed isolation of fibroin gene sequences; however, due to the limited amount of silk production by *T. urticae* and its fine structure, it was not possible to characterize this promising biomaterial. To overcome the limitation of low silk production rate, we established a culture of the related species, gorse spider mite, *Tetranychus lintearius*, that produces copious amounts of silk (*Tl-*S) (Fig. 1A), and using semi-industrial production generated a sufficient amount of silk for biochemical and physical characterization. We have shown that *T. lintearius* silk has thickness and physical characteristics similar to *T. urticae* silk. We characterized native *Tl-*S and produced nano-particles generated from *Tl-*S (*Tl-*SN) and compared then to *Bombyx mori* silkworm native silk fibroin (*Bm-*SF) and nanoparticles (*Bm-*SFN) as the standard in the field. Exposure of mouse fibroblasts to *Tl-*S-derived biofilm and nanoparticles demonstrated that this cell line can grow successfully in culture at a comparable level to that seen in the presence of *Bm*-SF derived nanoparticles and biofilm. Finally, we fluorescently labelled *Tl-*SN and showed that they can enter the cytoplasm of cultured cells. These experiments suggest that *Tl*-S is a new cytocompatible material and a potential source of natural nanoparticles with potential for various applications, including pharmacology and biomedicine.

## Results

### *T. lintearius* silk has nano-scale thickness and high Young’s modulus

*T. lintearius* produces copious amounts of silk, an order of magnitude higher than other *Tetranychid* mites (Fig. 1A). *T. lintearius* adults (Fig. 1B), nymphs and larvae synthetize silk continuously throughout their life, wrapping gorse cuttings in our production modules with a dense silk cover reminiscent of cellophane wrap. The native silk collected from the plant and visualised by scanning electron microscopy (SEM) shows a dense, woven structure of silk threads of various sizes (Fig. 1C). When we allowed mites to spin silk with lower density in petri-dishes, we were able to measure individual silk threads (Fig. 1D,E) that showed various thicknesses, representing multiple deposition of silk fibres as in *T. urticae*^27^, with even the thickest fibres below the micron range. To measure the thickness of single newly-spun fibres, we placed *T. lintearius,* both adults and larvae, on a silicon grid (Fig. 1F) and imaged the resulting fibres using an atomic force microscope (AFM) as described by Hudson et al*.* in 2013^26^.

**Figure 2:**
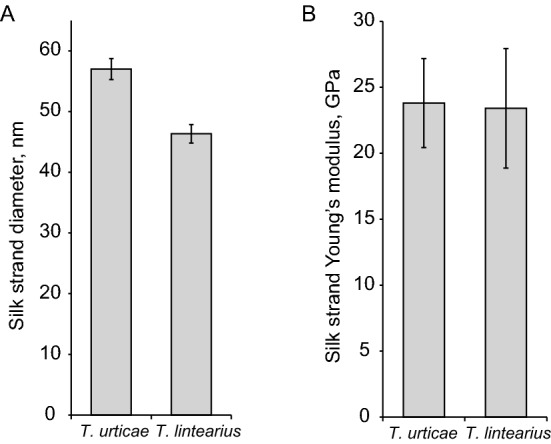
*T. lintearius* individual silk fiber thickness and Young’s modulus compared with silk of *T. urticae*. (**A**) Silk fiber thickness of *T. urticae* and *T. lintearius* measured by AFM; (**B**) Young’s modulus of individual silk fiber of *T. urticae* and *T. lintearius* measured by AFM. Data shown are mean ± SEM (*n* = 7).

The thickness of individual newly-spun *T. lintearius* silk (*Tl-*S) fibres was 45 nm, slightly thinner than *T. urticae* silk fibre thicknesses measured previously^13^ (Fig. 2A). We determined the Young’s modulus of individual *Tl-*S fibres to be in the range of 20 GPa (Fig. 2B), similar to that previously measured for *T. urticae* silk using the same methods^13,26^.

### Characterization of native *T. lintearius* silk

**Figure 3:**
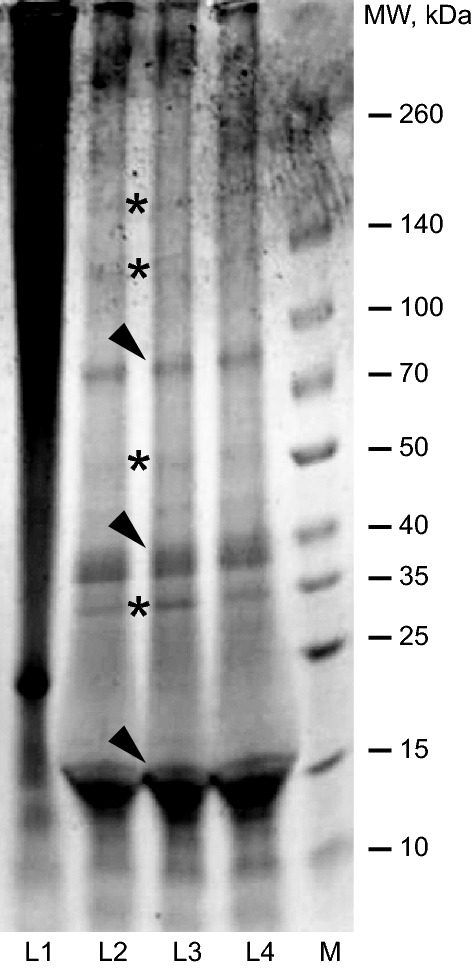
SDS-PAGE gel of regenerated silk fibroin from *B. mori* (L1) and silk from *T. lintearius* (L2, L3, L4). Arrowheads mark highly stained bands at 15 kDa, 40 kDa and 85 kDa and asterisks indicate moderately stained bands at 35 kDa, 50 kDa, 140 kDa and ≈ 200 kDa.

Sodium dodecyl sulphate–polyacrylamide gel electrophoresis (SDS-PAGE) was performed in order to determine the molecular weight of the peptides obtained from the *T. lintearius* and *B. mori* silks. After staining with Coomassie Brilliant Blue (Fig. 3) the native silk proteins appear as a partly degraded mixture of polypeptides of different molecular weights, concordant with our previously published results for regenerated fibroin from *B. mori* dissolved by LiBr^28^. In *B. mori* (Fig. 3, lane 1) we detected a band corresponding to the L-chains of silk fibroin (SF) at the position around 25 kDa. A region intensely stained at the upper position of the gel corresponds to H-chains of SF (350 kDa), as well as degradation peptides from H-chains, as a result of the processing protocol (see “Methods” section).

The three replicates of silk solutions from *T. lintearius* showed a similar band pattern (lanes 2, 3 and 4 of the gel), confirming the reproducibility of the processing methodology. Three highly stained bands at around 15 kDa, 40 kDa and 85 kDa are revealed. Other weaker bands appeared at 35 kDa, 50 kDa, 140 kDa and ≈ 200 kDa. The identity of these bands should correspond to the less degraded *Tl-*S peptides.

### Preparation of silk nanoparticles

The ability of spider mite silk fibroins to assemble into nanoparticles is comparable to that of silkworm silk fibroin, but particles are coloured mainly due the pigments that were present in the starting protein solution although they were partially lost during the washing steps. Nanoparticles were insoluble in water and the alcohols used during the preparation protocol, in agreement with the properties previously reported for similar silk nanoparticles^29–33^. In order to perform flow cytometry and cell penetration studies, a FITC labelling protocol was applied to the nanoparticles^34^, giving an intense yellow colour to the nanoparticles and intense fluorescence under ultraviolet light (λ_ex_ = 365 nm). After a freeze-drying process, the nanoparticles were collected as a pale brown fine powder, which can be easily dispersed in water by slight sonication to reconstitute a homogenous dispersion of nanoparticles.

### Attenuated total reflectance Fourier transform infrared spectroscopy reveals native silk and nanoparticle structure

**Figure 4:**
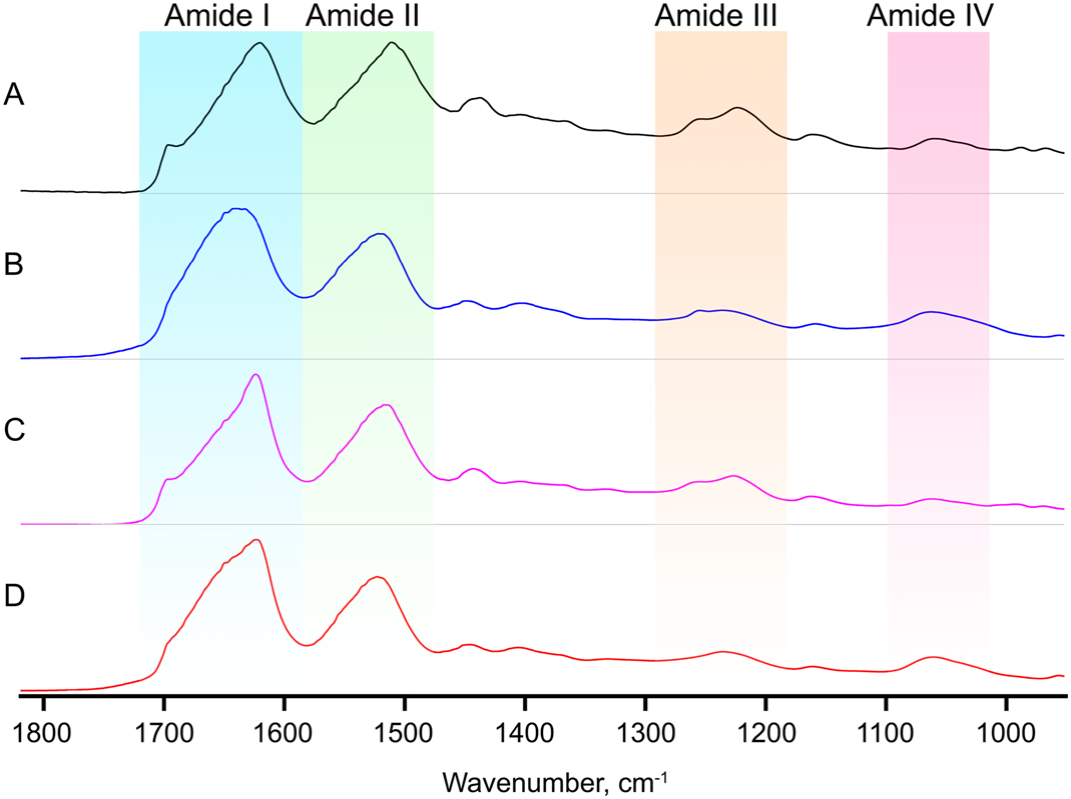
Amide region (I–IV) of the spectra of: (**A**) silk from *T. lintearius*; (**B**) degummed fibroin from *B. mori*, and (**C**) and (**D**) the nanoparticles obtained from them, respectively.

Infrared spectra of the native silks and the derived nanoparticles were recorded in order to determine the structural conformations of the proteins (Fig. 4) and identify possible differences between the raw biomaterials and their processed products.

The nanoparticles produced from *B. mori* silk fibroin (*Bm-*SFN) exhibited the characteristic peaks of silk fibroin amide I (1626 cm^−1^), amide II (1516 cm^−1^), amide III (1231 cm^−1^) and amide IV (1067 cm^−1^) in the β-sheet conformation^35,36^. The absence of a peak at 1660 cm^−1^ indicates that the conformation of the protein changed from random coil to β-sheet^37^; this change indicates an effective transition from a water-soluble to a non-soluble state. The results obtained from the particles produced from *Tl*-S revealed similar peaks to at 1622 cm^−1^, 1520 cm^−1^, 1237 cm^−1^ and 1065 cm^−1^. As seen by the infrared spectra in Fig. 4, no significant structural differences exist between the nanoparticles produced from the two different silks.

**Figure 5:**
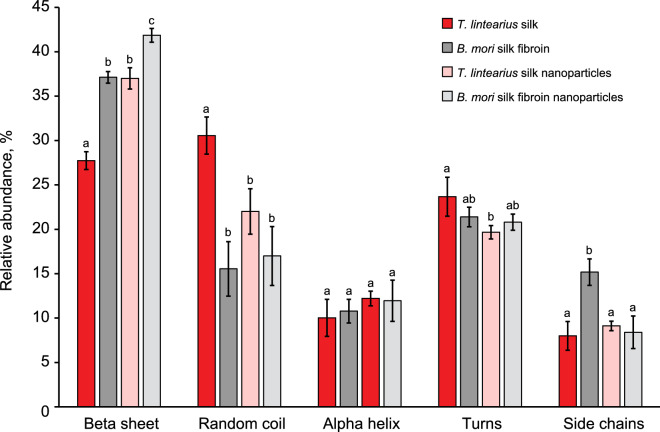
Secondary structures calculated from analysis of the IR spectra covering the amide I region (1735–1580 cm^–1^) of freeze dried silk from *T. lintearius*, degummed silk fibroin from *B. mori*, and the nanoparticles obtained from them. Data are expressed as mean ± SD (*n* = 3). Groups denoted by different letters are statistically different (*p* < 0.05).

Fourier self-deconvolution (FSD) of the infrared spectra covering the amide I region (1580–1760) was performed on native silk from *T. lintearius* (*Tl*-S) and degummed silk fibroin from *B. mori *(*Bm-*SF) as well as their corresponding nanoparticles in order to quantify the different secondary structures in the samples^38^. The relative content of the secondary structures showed a significantly lower percentage of β-sheet (*p* < 0.05) in the *Tl-*S and *Tl-*SFN relative to their *B. mori equivalents* (Fig. 5).

On one hand, *Tl*-S presented the highest proportion of random coil structures. On the other hand, *Bm*-SFN showed the highest percentage of β-sheet of all samples, indicating that most of the small peptide chains, which were mostly in random coil conformation, had been lost during the formation of the nanoparticles.

### Structure and characterization of *T. lintearius* silk fibroin nanoparticles

**Figure 6:**
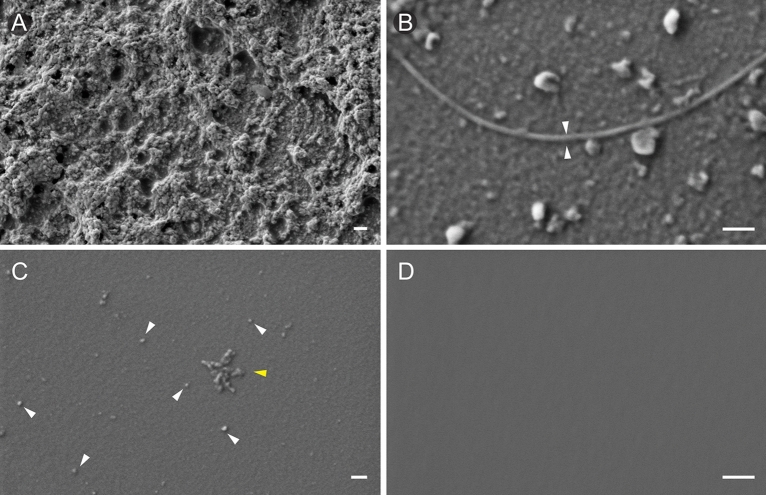
Focused Ion Beam milling combined with Scanning Electron Microscopy (FIB-SEM) of *T. linteariu*s nanoparticles at different concentrations. (**A**) 10X dilution of *T. lintearius* nanoparticles showing dense conglomerates of particles. (**B**) 100X dilution shows spontaneous formation of silk fibers (arrowheads). (**C**) At 1000X dilution individual nanoparticles show diameter of ca. 20 nm (arrowheads) (**D**) Silicone wafer with no sample loaded (control). Scale bar: a—200 nm, b–d—100 nm.

Focussed Ion Beam milling combined with Scanning Electron Microscopy (FIB-SEM) was performed on nanoparticle samples in different dilutions (see “Methods” section) after failing to obtain satisfactory images by conventional SEM. At low dilution, nanoparticles formed dense agglomerates (Fig. 6A). At still lower dilutions, we observed a uniform “film” of individual nanoparticles and spontaneous formation of fibres of ca. 20 nm in diameter (Fig. 6B). At a dilution of 1 µg/mL, individual round nanoparticles of ca. 20 nm were visible (Fig. 6C, arrowheads). To exclude the possibility that these features were artefacts of the wafer structure, we imaged unloaded wafers which showed no structures (Fig. 6D).

Following the FIB-SEM study, in order to investigate the stability and the hydrodynamic behaviour of the nanoparticles of *T. lintearius* and their analogues from *B. mori*, aqueous suspensions of the raw nanoparticles and their FITC-labelled derivatives were studied by Dynamic Light Scattering. Dispersions of *Tl*-SN and *Bm*-SFN in ultrapure water yielded a narrow mono-modal size distribution with an average diameter (*Z*_ave_) in the range 116–141 nm, polydispersity index (PdI) lower than 0.16 and negative values of ζ-potential in all cases (see Table 1). The value of *Z*_ave_ for the *Tl*-SN samples was slightly lower than for the *Bm*-SFN. The *Z*_ave_ of the FITC-labelled nanoparticles was 11 nm higher for the FITC-*Tl*-SN relative to *TI*-SN, while an increment of only 6 nm was seen for FITC-*Bm*-SFN relative to *Bm*-SFN. All values of polydispersity were similar (PdI ~ 0.150). As previously described, FITC-*Bm*-SFN can be considered equivalent to non-labelled *Bm*-SFN in terms of morphological characteristics^34^. The ζ-potential of the samples were significantly negative in all cases, but shifted toward positive values from –27.2 ± 1.5 mV for *Tl*-SNs to –21.4 ± 1.0 mV for FITC-*Tl*-SN, and from –30.0 ± 0.1 mV for *Bm*-SFNs to –25.1 ± 0.7 mV for FITC-*Bm*-SFN, negative enough to stabilize the nanoparticle suspension through strong electrostatic repulsion.

**Table 1:** Summary of the characteristics of the nanoparticles obtained by Dynamic Light Scattering: nanoparticle size, ζ-potential and mobility.

Sample name	Z-Average (nm)	Polydispersity index	Zeta potential (mV)	Wall zeta potential (mV)	Mobility (m^2^/V·s × 10^–8^)
*Tl*-SN	116.0 ± 0.9	0.142 ± 0.008	− 27.2 ± 1.5	− 35.4 ± 1.5	− 2.132 ± 0.119
FITC-*Tl*-SN	127.1 ± 0.2	0.154 ± 0.011	− 21.4 ± 1.0	− 22.6 ± 0.9	− 1.675 ± 0.085
*Bm*-SFN	135.2 ± 1.5	0.142 ± 0.010	− 30.0 ± 0.1	− 37.7 ± 1.4	− 2.356 ± 0.012
FITC-*Bm*-SFN	141.3 ± 1.7	0.158 ± 0.010	− 22.0 ± 0.6	− 25.1 ± 0.7	− 1.727 ± 0.049

Values presented as mean ± standard deviation (n = 3).

### Cytocompatibility/cytotoxicity evaluation of *T. lintearius* and *B. mori* biofilm and nanoparticles

**Figure 7:**
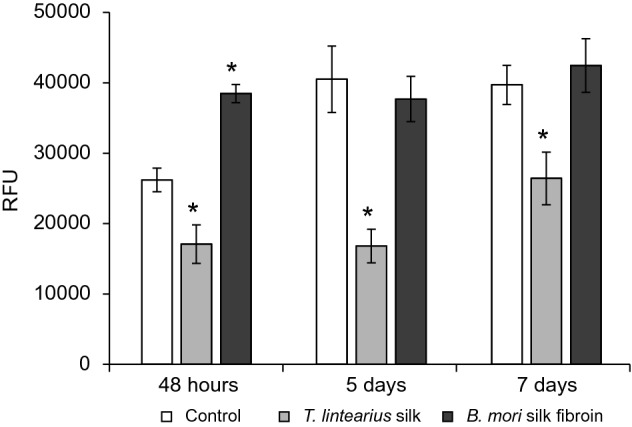
Proliferation of the L929 fibroblast cell line growing on the *T. lintearius* silk (*Tl*-S) and *B. mori* silk fibroin (*Bm*-SF) films at 48 h, 5 days and 7 days after seeding. The control corresponds to cells growing on the nude culture plates. Data are expressed as the average values of relative fluorescence units (RFU) (570–610 nm) ± SD (*n* = 5) of the PrestoBlue test. (*) represents significant statistical differences relative to group control.

Although previous studies have revealed the favourable biocompatibility of silk fibroin from *B. mori* and its excellent properties for use as a biomaterial in tissue engineering applications^39–41^, no cytocompatibility or biocompatibility studies are available for *Tl-*S, nor for any silk derived from mites. To test *Tl-*S cytocompatibility, we performed a study with silk films and nanoparticles of *T. lintearius* using the murine fibroblast cell line L929, which is commonly used in the evaluation of cytocompatibility and cytotoxicity of potential biomedical devices^42^. We deposited thin films of *Bm*-SF and *Tl*-S from their corresponding aqueous dissolution onto polystyrene culture plates and investigated the proliferation of L929 cells seeded on the films, as determined by a PrestoBlue assay. In addition, cells growing onto the nude polystyrene culture plates were use as a reference control of the cellular proliferation rate. The results of the proliferation test at 48 h, 5 days and 7 days after cell seeding on the silk films are shown in Fig. 7. We observed a lower proliferation rate of L929 fibroblasts on *Tl*-S films compared to cells growing on the control polystyrene wells and *Bm*-SF films; nevertheless, this lower cell proliferation on *Tl*-S films represents a rate of 65–66% at 48 h and 7 days of study with respect to the optimal conditions for cell culture of the control wells. It appears that cells took a little longer to settle down and adhere to *Tl*-S films before they began to grow and proliferate since relative fluorescence units of PrestoBlue assay with *Tl*-S films increased at the end of the study (Fig. 7), pointing to non-cytotoxic or cytostatic effects of *Tl*-S films.

**Figure 8:**
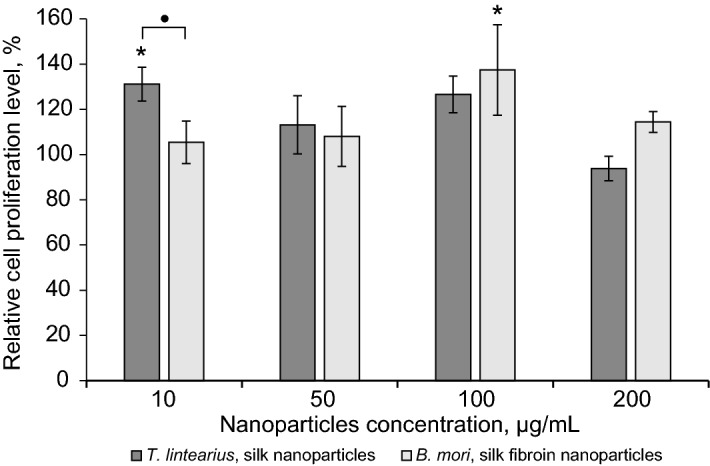
Cell proliferation of the L929 cell line for aqueous dispersions of *Tl*-SN and *Bm*-SFN at different concentrations, based on the PrestoBlue assay. Data are expressed as mean ± SD (*n* = 4). (*represents significant statistical differences between different nanoparticles concentration data and controls (100% proliferation), while significant statistical differences among different studied silk types are indicated by the (•) symbol.

*Tl-*SN were also evaluated in vitro by exposition of the L929 cell line to different concentrations of 10, 50, 100 and 200 µg mL^−1^ of *Tl*-SN in the culture medium. We evaluated cell proliferation in the presence of *TI*-SN compared with the values in the control wells (no *Tl*-SN added to the culture). As can be seen in Fig. 8, the concentration of 10 µg mL^−1^ *Tl*-SN induced 30% superior cell proliferation relative to both control and *Bm*-SFN.

Concentrations of 50, 100 and 200 µg mL^−1^ of *Tl*-SN showed a statistically similar cell proliferation relative to cells growing in DMEM expansion medium (control) and comparable values to those exposed to same concentrations of *Bm*-SFN, previously reported as good candidates for drug delivery or functionalization by diverse compounds with anti-inflammatory, anti-oxidant or anti-tumour properties, among others^29,32,34,43,44^. These *Tl*-SN results, together with those obtained from *Tl-*S films indicate a good cytocompatibility and no-cytotoxicity of the *Tl*-S.

### Nanoparticle cellular uptake

**Figure 9:**
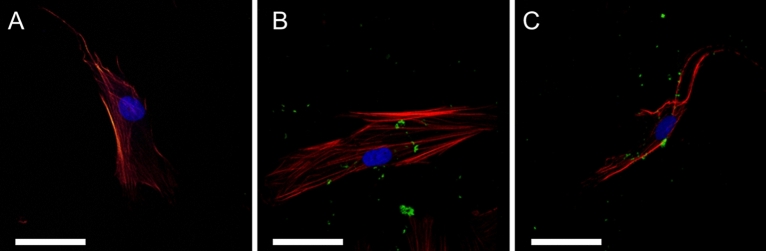
Confocal laser scanning microscopy of HDF cell line after 24 h of exposure to FITC-labelled silk nanoparticles (green). (**A**) Control cells grown without NP exposure; (**B**) HDF + *Tl*-SN; (**C**) HDF + *Bm*-SFN. Nuclei were stained with DAPI (blue) and cytoplasmic actin filaments with Atto Rho6G phalloidin (red). Scale bar: 50 µm.

To test the potential of *Tl*-SN as possible carriers of pharmacological molecules, we examined cellular localization of fluorescently labelled *Tl*-SN applied to HDF and HepG2 cells. These cell lines were chosen as representative of a normal and healthy tissue, in the case of HDF cells that are derived from the dermis of human skin, or tumour cell tissue, with respect to HepG2 of hepatocellular carcinoma origin. In both cases, cellular uptake of FITC-labelled nanoparticles (NP) was observed by confocal laser scanning microscopy (Fig. 9), with clusters of FITC-labelled *Tl*-SN and *Bm*-SFN in green (Fig. 9B,C, respectively) associated with cytoplasmic actin filaments (red).

**Figure 10:**
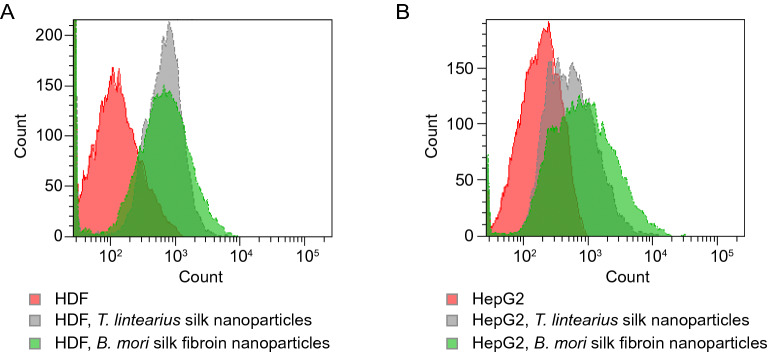
Detection and quantification by flow cytometry of HDF and HepG2 cellular uptake of FITC-labelled nanoparticles of *T. lintearius* silk (*Tl*-SN) and *B. mori* silk fibroin (*Bm*-SFN). Flow cytometry histograms of cell count versus FITC intensity of the studied FITC-labeled NP added to (**A**) HDF and (**B**) HepG2 cells after 24 h of cell exposure.

The cellular uptake was quantified by determining the median intensity of the internal fluorescence (FITC) signal in the populations of HDF and HepG2 studied using flow cytometry. After culture exposure to 50 µg mL^−1^ of both *Tl*-SN and *Bm*-SFN, median cell fluorescence intensity values increased relative to controls, suggesting that FITC-labelled NP have been successfully internalized by cells (Fig. 10).

*Tl*-SN and *Bm*-SFN showed distinct uptake in different cell lines (Fig. 10). In HDF cells, *Tl*-SN and *Bm*-SFN showed similar fluorescence values, however rSD fluorescence variation was much larger in *Bm*-SFN treatment showing that *Tl*-SN were more uniformly internalized in this cell line (Table 2). In HepG2, *Bm*-SFN treatment induced higher fluorescence than *Tl*-SN. These findings confirmed the internalization of both FITC-labelled nanoparticles into HDF and HepG2 cell lines and showed a cell-line specific internalization pattern, suggesting the possibility for various applications of *Tl*-SN and *Bm*-SFN depending of specific cell population.

**Table 2:** Median fluorescent intensity and rSD values of the FITC signal detected in HDF and HepG2 cell populations after 4 h and 24 h of cell exposure to FITC-labelled *Tl*-SN and *Bm*-SFN.

	Median fluorescence		rSD	
HDF	110		84	
HepG2	177		122	

	4 h	24 h	4 h	24 h
HDF + *Tl*-SN	302	670	166	391
HDF + *Bm*-SFN	317	698	217	522
HepG2 + *Tl*-SN	342	459	217	332
HepG2 + *Bm*-SFN	440	695	355	624

## Discussion

In this study, we have characterized for the first time structural and biological features of the silk from acari, spider mite *T. lintearius*, including its mechanical characteristics and protein structure. Furthermore, we derived nanoparticles and biofilm from this natural nano-silk and showed that this material stimulates cell proliferation and does not exhibit cytostatic or cytotoxic effects. Finally, *Tl-*S nanoparticles demonstrate an ability to be internalized in tumour cell lines, showing a potential for use as natural drug carriers in biomedicine.

The nanometre-scale diameter of spider mite silk and its extremely high Young’s modulus was known from the silk fibres produced by the two-spotted spider mite (TSSM), *T. urticae*^13,26^. The diameter of *Tl-*S is slightly lower than that of TSSM and its Young’s modulus in the 20 GPa range places this silk together with bagworm silk in a group of natural silks with high Young’s modulus^17^. This silk has double the Young’s modulus of all spider silks such as *Araneus diadematus*, *Nephilla clavipes*, *Latrodectus hesperus*, and *Caerostris darwini*, which were considered to be the most attractive natural silks in terms of their mechanical properties. Silk in spider mites evolved independently from spiders both in their manner of production and their application. In spider mites, silk is produced from labial silk glands (in a manner similar to insect silks), in contrast to spider silk production from the abdominal spinneret gland.

The nanoscale-dimension and high Young’s modulus of spider mite silk result from a gene sequence, known from the *T. urticae* genome, that probably evolved to provide multiple uses, such as mobility, protection from predators, climate control, formation of a substrate for egg laying, and pheromone communication.

To understand the structure of *T. lintearius* silk, we compared it to *B. mori* silk, whose structure is well known. In gel electrophoresis, native *T. lintearius* and *B. mori* silks show different band patterns following LiBr dissolution. While *B. mori* silk shows a simple pattern corresponding to light and heavy fibroin chains, *Tl*-S shows a more complex pattern, with multiple highly-stained bands ranging from 85–15 kDa and less strongly stained bands from 200–35 kDa. *B. mori* silk fibroin is composed mainly of three proteins: the fibroin heavy chain (FibH, 350 kDa), the fibroin light chain (FibL, 26 kDa), and the integrity-maintaining glycoprotein P25 (about 30 kDa)^45,46^. This difference may be the result of different structures of fibroin proteins of *T. lintearius*, differences in fibroin gene numbers, or a difference in fibroin degradation upon LiBr treatment. Protein patterns of dissolved *N. claviceps* silk also show a complex pattern producing multiple bands ranging from 320 kDa to small degraded polypeptides of 20 kDa and below. A similar more complex pattern is seen in barnacle adhesive secretions which exhibit elements of fibroin structure with multiple bands in range from 250–14 kDa^15^.

In order to explain differences in their mechanical characteristics, we compared *Tl-*S to *Bm-*SF using attenuated total reflectance Fourier transform infrared spectroscopy (ATR-FTIR). The native *T. lintearius* and *B. mori* silks showed structural differences with a higher proportion of β-sheets in *B. mori* native silk, while *Tl*-S contained a higher proportion of random coils. The same trend was reflected in the structure of nanoparticles produced from these silks. In principle, β-sheets contribute to fibroin strength, so it remains unclear how *Tl*-S with a relatively low content of β-sheet structure has a higher Young’s modulus. In *B. mori*, many factors influence the mechanical properties of silk fibres, including their diameter, sericin weight content and protein secondary structures. However, it is believed that the mechanical properties of silk are primarily dependent on protein structure. This is confirmed by both experimental and simulation results showing that the strength and stiffness of silk are determined mainly by β-sheet structure^47^, while the extensibility and toughness of silk are governed mainly by the semi-amorphous matrix^48–50^. Mechanical properties of silk fibres are therefore determined by the proportion of the semi-amorphous matrix, commonly in random coil conformation, and β-sheet crystallites. It is well known that the β-sheet nanocrystals serve as molecular cross-links that provide silk fibres with their great stiffness, while the amorphous regions play the opposite role and are responsible for their superb elasticity^51,52^. So far, there has been no reported experimental research to correlate the size of β-sheet crystallites to the mechanical properties of silkworm silk fibroin fibres. However, recent modelling of beta sheet structures found that β-sheet crystallites that are confined to a few nanometres can achieve higher strength, stiffness and toughness than larger β-sheet crystallites^53^. Thus, one possible explanation for the superior Young’s modulus of *Tl*-S could be in the sequence and size of β-sheet domains that are reduced by confinement in these natural nano-silk fibres.

Although *T. lintearius* silk nanoparticles in aqueous solution have a hydrodynamic radius similar to that of *B. mori* silk fibroin nanoparticles (~ 100 nm), in a dry state, *Tl*-SN had a spherical shape with a typical diameter of 20 nm, as visualized by FIB-SEM, five times smaller than in the aqueous state. At lower concentrations, we observed spontaneous formation of nanofibres (Fig. 6B), suggesting that these nanoparticles could interact between themselves and form short fibres, a performance of silk nanoparticles previously described^54^, and attributed to a silk fibroin assembly process that relies on the equilibrium between two opposing forces: the repulsion of negative charges on the surface of the protein aggregates or particles and the high affinity of the external surfaces. The fibrous structure shown in Fig. 6B suggests an asymmetric charge distribution on the protein surface at very high dilutions which allows polymerization of discrete areas of the peptide chains with the lowest electrostatic repulsion and the highest affinity. The anisotropy of the β-sheets can promote these arrangements. At higher concentrations, their strongly negative ζ values confer to the nanoparticle suspension a high stability, and no aggregation or sedimentation were observed in 1 mg mL^−1^ suspension for several months under refrigeration (4 °C).

One potential application of spider mite silk as a natural nanomaterial is in biomedicine, where natural silk-based nanoparticles have a wide range of applications including cancer therapy^55^, targeted drug delivery^56^ and development of various prosthetic devices^57^. Experiments performed so far on natural silks, including spider, *B. mori* and honeybee silk have demonstrated good biocompatibility^16^. However, the silk of some species, such as the moth *Antheraea pernyi*, has a cytotoxic effect on cells in culture^58^. No prior study exists on spider mite silk biocompatibility and potential cytotoxic effects to mammalian cells. To assess how *T. lintearius* silk affects cell growth, we performed a comparative study of mammalian cell proliferation in the presence of *T. lintearius* and *B. mori* silk biofilm and nanoparticles. Cytotoxicity was not detected in either biofilm or in nanoparticle-containing solution. Cell proliferation was higher on *B. mori* biofilm, but *T. lintearius* nanoparticles resulted in higher proliferation of mouse fibroblasts compared to the control and *B. mori* nanoparticle treatment, suggesting that this nanomaterial enhances cell proliferation in culture without negative effects of cytotoxicity.

One of the key questions for biomedical application, besides biocompatibility, is whether *T. lintearius* nanoparticles can enter the cell to deliver potential pharmacological agents. Target tissue accumulation is dependent on the physicochemical properties of the nanomedicine, and the size, shape, and surface chemistry of the nanoparticles, which govern their uptake into target sites^59–62^. The size and characteristics of the drug delivery particles will dictate the in vivo localization and avoidance of clearance mechanisms following administration, with a lower limit of ca. 5 nm to avoid renal filtration, and an upper limit of 200 nm to avoid excessive liver and spleen accumulation^63,64^. The mechanism of cellular uptake of foreign particles is determined by particle size^65^. Particles smaller than 200 nm enter via clathrin-mediated endocytosis, while the uptake of larger particles is mediated by a calveole internalization mechanism. Our quantitative fluorescence data and determination of sub-cellular localization of *T. lintearius* and *B. mori* nanoparticles demonstrate that both *T. lintearius* and *B. mori* nanoparticles enter efficiently into human dermal fibroblasts and hepatocellular carcinoma cell lines. However, it is unclear whether these clusters represent endosomes that are involved in particle internalization or represent localization of internalized particles to lysosomes. Taken together with positive effects to cell proliferation and lack of cytotoxicity, this demonstrates that *T. lintearius* nanoparticles can serve as agents for delivery of pharmacological agents to the cell.

This study represents a promising first step for further development of spider mite silk as a sustainable and valuable new source of nanomaterials. The genome sequence of *T. urticae* determined recently and ongoing genome sequencing of *T. lintearius* using long-range sequencing by Oxford Nanopore have the potential to reveal the full complement of fibroin genes in this group of silk-spinning mites. Outstanding genomic resources and small genomes in these species provide a potential for recombinant silk production and open the possibility of developing functionalized nanomaterials using active targeting as well as a better understanding of silk structure and polymerization.

## Methods

### Silk production

*T. lintearius* was reared on its natural host *Ulex europaeus* at the ICVV, (Logroño, La Rioja), in a rearing chamber at a temperature of 24 °C, with a 16 h light/8 h dark cycle, and humidity of 60%. Rearing modules consisting of *U. europaeus* plant cuttings placed in tube racks in tray filled with water were used for silk mass production (Fig. 1). Silk was collected from the plants using a toothpick (silk is wrapped on the toothpick in a similar manner as sugar wool, since threads adhere to each other) and used for nanoparticle preparation. Silk cocoons (SC) were obtained from *B. mori* silkworms reared in the sericulture facilities of IMIDA (Murcia, Spain) and raised on a diet of fresh natural *Morus alba* L. leaves. Cocoons were then stifled to kill the pupae by means of dry heat (85 °C)^66^. The intact chrysalides were extracted manually from the cocoons prior to silk processing and degummed in a boiling aqueous solution of Na_2_CO_3_ 0.05 M for 30 min in order to remove the sericin^67^. The silk fibroin fibres were further rinsed with ultrapure water and dried at room temperature overnight.

### Silk processing

The silk produced by *T. lintearius* was manually collected from gorse cuttings using toothpicks, containing at the end silk and other materials derived from the life cycle of spider mites. Three batches of 3.5 g of this material were dissolved in 9.3 M LiBr solution at 60 °C for 2 h following the previously described method^28^. Then the resultant dissolutions were dialyzed against distilled water for 3 days to remove the LiBr, filtered through Miracloth paper, and centrifuged to remove the insoluble fragments and dust. After this last step, the dissolutions were concentrated by dialysis against PEG (10,000 Da) for 9 h to obtain three replicates of aqueous *Tl*-S (1.3% w/v) and stored at 4 °C until use. *B mori* silk fibroin fibres were processed using the same protocol.

### Preparation of silk films

After processing, silk films were obtained by casting 230 µL of 1.3% (w/v) *Tl*-S aqueous dissolution per well (3 mg·well^−1^) on a 48-well tissue culture plate. Once dried at room temperature, these films were annealed with methanol (20 min) to produce water insoluble films. In order to test their cytocompatibility, *Tl*-S films were sterilized by immersion in ethanol 70% (v/v) for 10 min and washed twice with sterile 1 × PBS dissolution.

### Preparation of silk nanoparticles

In order to compare the ability of *T. lintearius* silk to assemble into nanoparticles compared with the silk fibroin from *B. mori,* we followed the protocol for the preparation of *T. lintearius* silk nanoparticles (*Tl*-SN) based on the method described previously by Lozano-Pérez et al.^32^ for *B. mori* silk fibroin nanoparticles, with modifications. Briefly, the freshly prepared *Tl*-S aqueous solutions (1.3 wt%) were slowly dripped (~ 1 drop every 2 s) in cold methanol while gently stirred. The methanol proportion in the final mixture was kept above 90% (v/v) in order to achieve an efficient conformation change of silk from random coil to β-sheet. After a few drops, a turbid amber-like suspension appeared, and the mixture was allowed to reach room temperature while stirring for 2 h. The particle suspension was then transferred to Falcon tubes and centrifuged at 10,000*g* for 15 min at 8 °C. Supernatant was discharged and an equal volume of dry methanol was added in order to wash pigments or contaminants adsorbed onto the particles. After sonication, centrifugation and decantation of the methanol supernatant, particles were washed (3x) with 30 mL of ultrapure water (18 MΩ·cm) and kept in water suspension at 4 °C until use or lyophilized for longer storage, yielding *Tl*-SN as light brownish powder or *Bm*-SFN as a white powder.

### Silk characterization

#### SDS-PAGE

The protein integrity of both *Tl-*S and *Bm*-SF in the aqueous solution were confirmed by SDS-PAGE. The SDS-PAGE was performed according to the Laemmli protocol^68^, with a 4–20% gradient acrylamide gel (Amersham GE-HC). The set-up used was a horizontal Gel-Box electrophoresis chamber (Amersham GE-HC). After electrophoresis, the gels were stained with 0.25% Coomassie Brilliant Blue (Acros Organics, Belgium). ColorBurst Electrophoresis Marker (Sigma-Aldrich, St. Louis, MO, USA) was loaded as molecular-weight markers. Protein concentrations in the samples were unified at 50 μg per lane by diluting the different protein aqueous solutions of *Tl*-S with ultrapure water (18 MΩ·cm). These samples were loaded under denaturing conditions by adding β-mercaptoethanol 10% (v/v) to the loading buffer and heating at 100 °C for 5 min immediately before the electrophoresis.

#### Atomic force microscopy

The Young’s moduli of silk nanofibers were measured by atomic force microscopy using a method previously described by Hudson et al^26^. Briefly, custom silicon substrates consisting of grids of trenches ~ 2 µm across and several 100 nm deep were prepared by photolithography and reactive ion etching. Silk fibres were suspended across the trenches by allowing adult and larval *T. lintearius* spider mites to traverse the substrates for several hours, after which suspended fibres were located by contact-mode imaging with NP-S silicon nitride cantilevers (Veeco Instruments) at low forces using a Veeco Instruments Multimode atomic force microscope (AFM) with Nanoscope IIIa controller.

Isolated suspended fibres were deformed by translating the sample stage vertically through ~ 1 µm at a rate of ~ 8 µm/s while measuring the resulting cantilever deflection over a grid of positions encompassing each fibre (i.e., by acquiring a force-volume image). This allowed transverse fibre deflection (the difference between the known vertical sample motion and the measured cantilever deflection) to be measured as a function of applied force (equal to the product of the cantilever deflection and its spring constant as determined by the thermal noise method)^69–72^. The resulting force vs. displacement curves at the centre of the suspended portion of each fibre were numerically analysed using the method of Hudson et al. to determine its Young’s modulus. All measurements were performed at a temperature of ~ 32 °C and relative humidity typically below 20%.

#### SEM

To visualise the nanoparticles, 20 µL of nanoparticle solution was applied to a silicon wafer in distilled water at dilutions of 10x, 100x, and 1000x. Following air-drying, 3 nm of Osmium coating was applied using a Filgen OPC80 Osmium Plasma Coater to make the sample conductive. The imaging was performed by a Zeiss/LEO 1540XB FIB/SEM using accelerating voltages of 1 keV with a 4 mm working distance.

#### Attenuated total reflectance Fourier transform infrared spectroscopy

Attenuated total reflectance Fourier transform infrared spectroscopy was used to analyse the structural features of *Tl-*S and *Bm*-SF or their corresponding nanoparticles. For each measurement, ~ 5 mg of dry sample was used without further manipulation. Each spectrum was acquired using a Nicolet iS5 spectrometer equipped with an iD5 ATR accessory (Thermo Scientific, USA) and controlled with OMNIC v.6.1.0.0038 software. Measurements were performed in absorbance mode with a resolution of 4 cm^−1^, a spectral range of 4000–550 cm^−1^, and 64 scans, using N-B strong Apodization and Mertz Phase correction. The analysis was finally focused in the range of 1700–1100 cm^−1^ as the most informative for the IR spectra of silk proteins.

Fourier self-deconvolution (FSD) of the infrared spectra covering the Amide I region (1735–1580 cm^–1^) of the spectra was performed and results were compared by means of OMNIC v.6.1.0.0038 software. Deconvolution was automatically performed by means of the function “peak resolve” using a Gaussian line shape. To measure the relative areas of the amide I components, FSD spectra were then curve-fitted. The positions (cm^–1^) of the band maxima in the deconvoluted spectra were made to correspond to the frequency of the minima in the second derivative of the undeconvoluted spectra. Finally, the deconvoluted amide I spectra were area-normalized. The relative areas of the single bands were used to calculate the fraction of the secondary structural elements. Vibrational band assignments were based on the data summarized by Hu et al. (2006)^38^.

#### Dynamic light scattering

The average size (hydrodynamic mean diameter or *Z*-average), size distribution (polydispersity index, PdI) and zeta potential (ζ) of nanoparticles were measured using a Malvern Zetasizer Nano ZSP instrument. For the determination of size distributions, samples were dispersed to a concentration of 0.5 mg mL^−1^ in ultrapure water via sonication (1 min at 10% amplitude using a Branson SFX-55Ò0 sonifier). All measurements were performed in water at 25 °C. The *Z*-average diameter, polydispersity and ζ values were calculated with the software provided by the manufacturer (Zsizer Software v.7.14) from the measurements performed in quintuplicate (12 runs/measurement).

#### Cellular in vitro assays

Every cell culture used in this work was tested for the absence of mycoplasma before performing the experiments and viability and cell number were determined by trypan blue staining using a Neubauer chamber. Culture growing conditions were 95% RH, 37 °C and 5% CO_2_. All the chemicals used for cell culture were purchased from Sigma-Aldrich (St. Louis, MO, USA) and Gibco (Paisley, UK); Nunc (Roskilde, Denmark) provided the culture plates.

#### Cytocompatibility/cytotoxicity assay

In order to analyse the potential use of *Tl*-S as a biocompatible material, *Tl*-S films and *Tl*-SN were produced and tested in vitro. Murine fibroblasts (L929 cell line, ECACC Nº 85011425) were chosen for the cell culture study as they are highly stable, fast growing and commonly used for cytotoxicity and cytocompatibility experiments. The L929 cells were seeded in 25 cm^2^ flasks at a density of 5·10^3^ cells·cm^–2^ in DMEM expansion medium (supplemented with 10% FBS, 100 U mL^–1^ penicillin and 100 μg·mL^–1^ streptomycin). Medium was carefully replaced twice per week and cells were allowed to grow until the culture reached 80% confluence.

To test the cytocompatibility of *Tl*-S films, the cells were detached using 0.05% trypsin/EDTA and seeded on the films at a density of at 5·10^3^ cells·cm^–2^ with 1 mL of DMEM expansion medium. As positive controls for adhesion and proliferation, tissue culture polystyrene substrates (TCPS) were also seeded in the same conditions. Cell proliferation was measured 48 h, 5 and 7 days after seeding using PrestoBlue (PB) reagent (Invitrogen, Thermo Fisher Scientific, Waltham, MA, USA), a resazurin-based membrane permeable solution which does not require cell lysis.

In the case of *Tl*-SN, L929 cells were seeded at a density of 15·10^3^ cells·cm^–2^ with 1 mL of DMEM expansion medium on a nude 48-well tissue culture plate and incubated 24 h to achieve cell adhesion and expansion. After this period, *Tl*-SN were added to the culture at 10, 50, 100 and 200 µg mL^−1^ to evaluate their potential cytotoxicity using the PB assay after 24 h exposure to the nanoparticles. For comparison purposes, samples from *B. mori* SF were also included in both film and nanoparticle experiments.

PB quantitatively analyses proliferation of metabolically active cells by mitochondrial reduction of resazurin to a red fluorescent compound called resorufin; as a consequence, the reagent exhibits a change in colour, as well as a shift in its fluorescence. Following the manufacturer’s protocol, a 10% solution of PB was added to the wells and incubated for 4 h at 37 °C in a 5% CO_2_ atmosphere. The solution was then removed and relative fluorescence (RF) was measured using a Synergy MX microplate reader (BioTek Instruments, VT, USA) and controlled with Gen5 v.1.08.4 software (BioTek Instruments, VT, USA) with an excitation wavelength of 570 nm and an emission wavelength of 610 nm.

#### Nanoparticle cellular uptake

Human dermal fibroblasts (HDF (106-05a), ECACC Nº 06090715) or hepatocellular carcinoma cell line (HepG2, ATCC HB-8065) were chosen to study cellular uptake of silk NP. HDF cells were cultured in DMEM/F-12 expansion medium (supplemented with 5% FBS, 100 U mL^–1^ penicillin and 100 μg mL^–1^ streptomycin), while HepG2 cells require EMEM expansion medium (supplemented with 10% FBS, 100 U mL^–1^ penicillin and 100 μg∙mL^–1^ streptomycin). Medium was carefully replaced twice per week and cells were allowed to grow until the culture reached 80% confluence, when they were detached using 0.25% trypsin/EDTA, seeded in 6-well plates at a density of 6·10^3^ and 20·10^3^ cells·cm^–2^ respectively and incubated 24 h to achieve cell adhesion and expansion. FITC-labelled *Tl*-SN or *Bm*-SFN at 50 µg mL^−1^ were added 24 h after seeding and cells were incubated at 37 °C. After 4 and 24 h of NP exposure, cells were washed with PBS 1x, trypsinized, and resuspended in culture medium. Flow cytometry was performed (LSRFortessa X-20, Becton Dickinson), quenching extracellular fluorescence by trypan blue, and the median fluorescence intensity of the cell population that internalized FITC-labelled NP was determined using the FACSDiva v.8.0.2 software (Becton Dickinson).

To confirm cellular uptake of nanoparticles, confocal laser scanning microscopy imaging was performed. 24 h after NP exposure, cells were washed twice with PBS 1 × and fixed for 10 min in 4% paraformaldehyde. Actin filaments and nuclei were respectively stained by 40 min of incubation with Atto Rho6G phalloidin and DAPI (Sigma-Aldrich (St. Louis, MO, USA)). Confocal images were obtained with a Leica TCS SP8 microscope and Leica Application Suite X (LAS X) v.3.5.5 software (Leica Microsystems AG, Germany).

### Statistical analysis

All analyses were performed with the statistical software IBM SPSS Statistics v.25. When data followed the normality and homogeneity of variance requirements, their means were compared by the parametric test ANOVA followed by Bonferroni’s post hoc multiple t-test. Significance level was set to *p* < 0.05.

## Supplementary information

Supplementary Legend.

Supplementary Video.
