# Supplementary material for: The silk of gorse spider mite Tetranychus lintearius represents a novel natural source of nanoparticles and biomaterials
Source: Sci Rep. 2020 Oct 28;10:18471. doi: 10.1038/s41598-020-74766-7 (PMC7595037; doi:10.1038/s41598-020-74766-7)
Supplement: Supplementary file 1 — Supplementary Legend. [file 41598_2020_74766_MOESM1_ESM.docx]

**SUPPLEMENTARY MATERIAL**

**for**

**The silk of gorse spider mite *Tetranychus lintearius*** **represents a novel natural source of nanoparticles and biomaterials**

Antonio Abel Lozano-Pérez*^1&^, Ana Pagán*^1^, Vladimir Zhurov^2^, Stephen D. Hudson^3^, Jeffrey L. Hutter^3^, Valerio Pruneri^4^, Ignacio Perez-Moreno^5^, Vojislava Grbic’^2^, José Luis Cenis^1^, Miodrag Grbic’^2,5,6&^, Salvador Aznar-Cervantes^1^

^1^ Departmento de Biotecnología, Genómica y Mejora Vegetal, IMIDA, c/ Mayor, s/n, 30150 La Alberca (Murcia), Spain

^2^ Department of Biology, The University of Western Ontario, London, Ontario N6A 5B7, Canada

^3^ Department of Physics and Astronomy, The University of Western Ontario, London, Ontario N6A 3K7, Canada

^4^ICFO—Institut de Ciències Fotòniques, The Barcelona Institute of Science and Technology, 08860 Castelldefels, Barcelona, Spain

^5^Dep. Agriculture and Food, University of La Rioja, c/ Madre de Dios, 53, 26006 Logroño (La Rioja), Spain

^6^ Department of Biology, University of Belgrade, Serbia

*Authors contributed equally to the work.

^&^ Corresponding authors


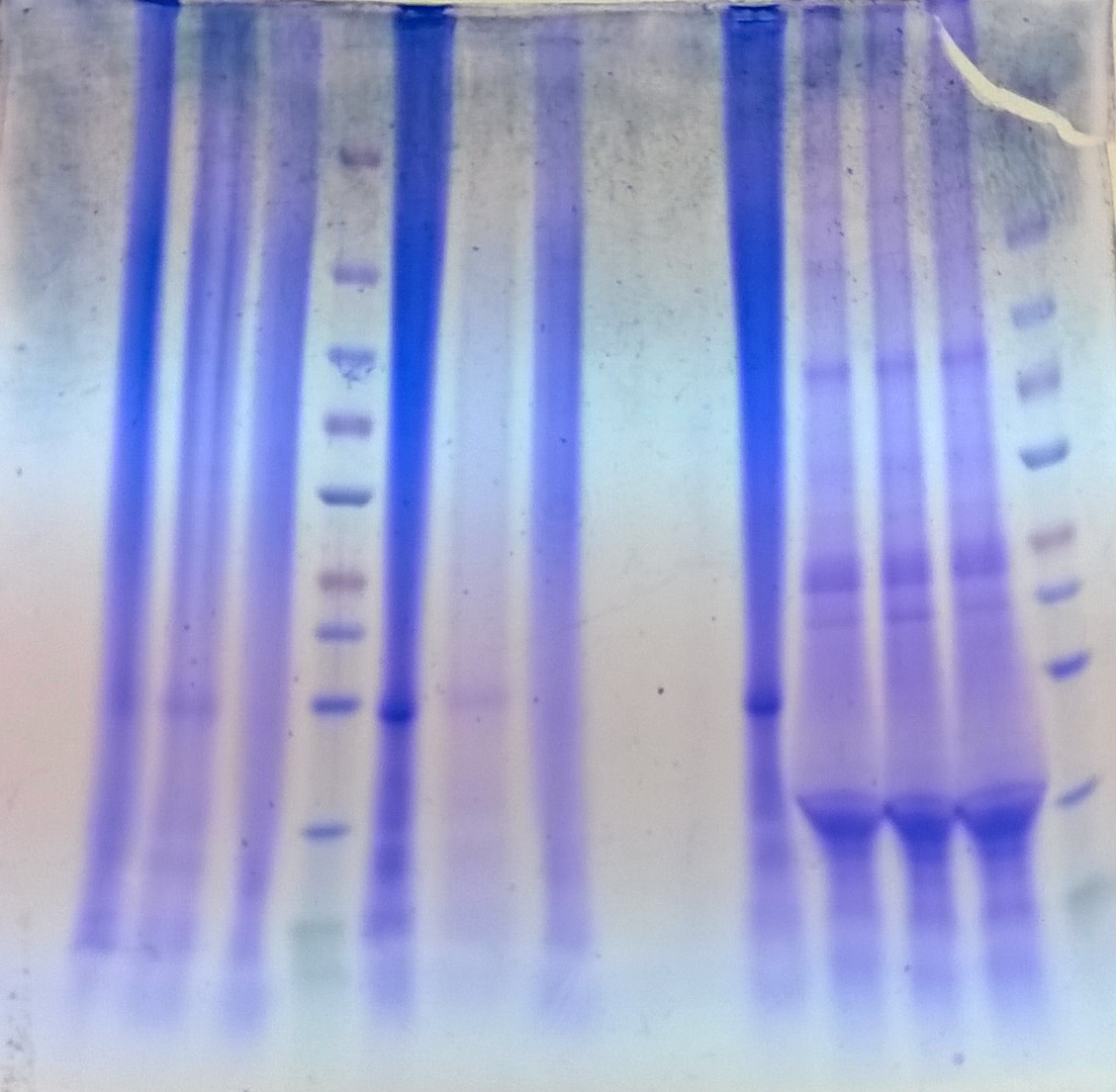
Figure S1. Original picture of the SDS-PAGE gel partially reproduced in Figure 3, in compliance with the digital image and integrity policies of the journal.

Supplementary Video. 3D animation from confocal laser scanning microscopy of internalized FITC-labelled *Tetranychus lintearius* silk nanoparticles (green) in HDF cell line after 24h of exposure. Nuclei were stained with DAPI (blue) and Atto Rho6G phalloidin stained cytoplasmic actin filaments (red).

Tl-SNs_cellular_uptake_3D.mp4
